# Hyper-dilute botulinum toxin as an effective treatment for refractory erythromelalgia

**DOI:** 10.1016/j.jdcr.2024.07.044

**Published:** 2024-09-19

**Authors:** Ariel Brown, Duaa AbdelHameid, Kaveh Nezafati, Heather W. Goff

**Affiliations:** Department of Dermatology, University of Texas Southwestern Medical Center, Dallas, Texas

**Keywords:** erythema, erythromelalgia, hyper-dilute botulinum toxin, onabotulinum toxin A, neuropathic pain

## Introduction

Erythromelalgia is a rare skin disorder considered a cutaneous manifestation of neuropathic pain and microvascular dysfunction and is characterized by intense cutaneous burning, warmth, and erythema.^1^^,^^2^ Mechanistically, symptoms are attributed to a gain-of-function mutation in SCN9A, a gene encoding the Nav1.7 sodium receptor that causes nociceptive neurons to become hyperexcitable and release substance P, neurokinin A, and calcitonin gene-related peptide (CGRP) neuropeptides. In addition, pro-inflammatory agents such as prostaglandins, leukotrienes, serotonin, bradykinin, histamine, and cytokines further sensitize the nociceptors.^1^ With numerous pain and inflammatory modulators involved in its pathophysiology, treatment often requires a multimodal, step-wise approach, and even still, some cases remain refractory.^3^

## Case report

**Fig 1:**
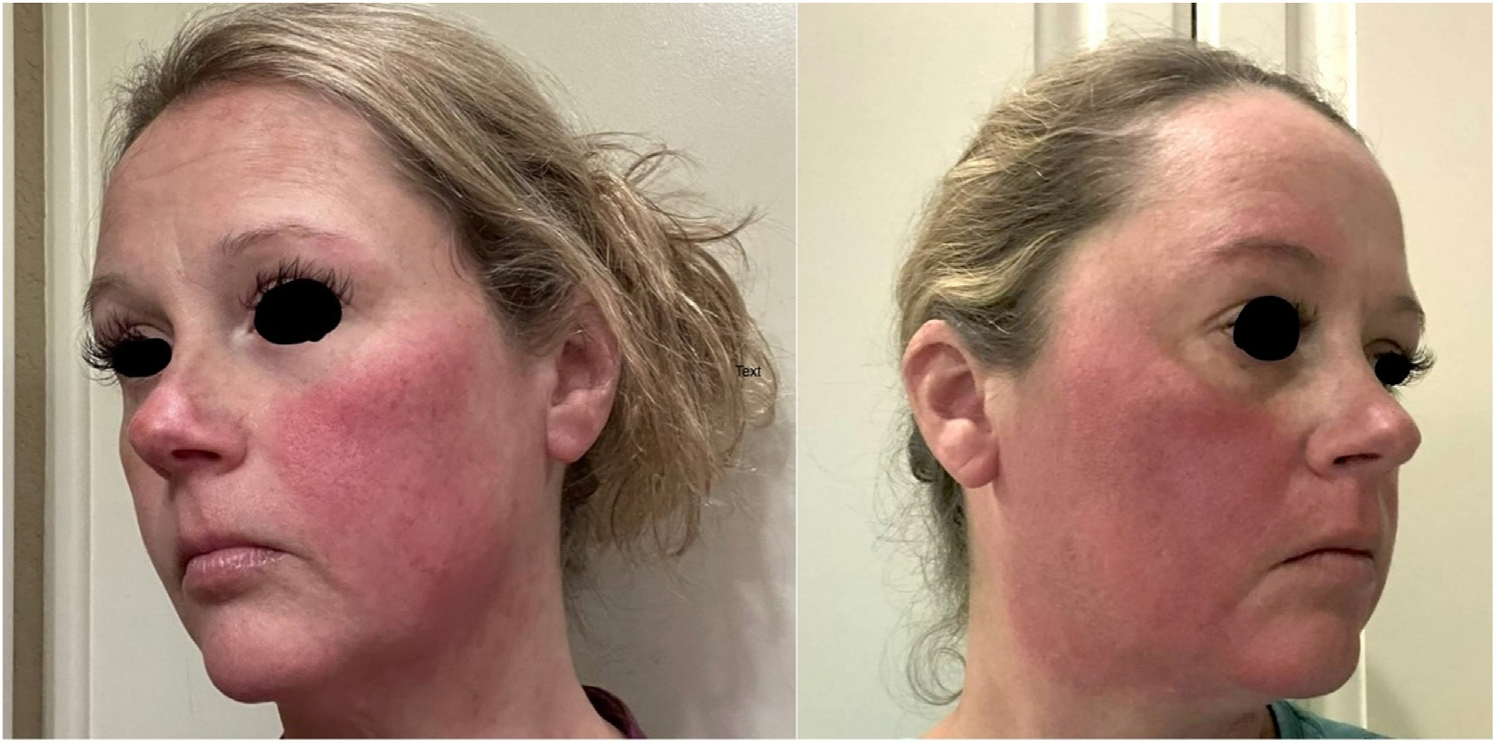
Two separate occasions of the patient with an active erythromelalgia facial flare.

An otherwise healthy Caucasian female patient in her 40s presented to our academic center for further management of an episodic burning rash present on her face and extremities for 10 to 15 years consistent with erythromelalgia. Her flares were occurring up to 4-5 times weekly. Prior failed treatments included several oral and topical agents: colchicine 0.6 mg twice daily, cromolyn 200 mg (10 mL) 4 times daily, hydroxyzine 250-300 mg daily, gabapentin 300 mg 3 times daily, and pentoxifylline 400 mg 3 times daily; compounded amitriptyline 2%/ketamine 1%; and compounded gabapentin 6% topical cream, none of which decreased the frequency or severity of her flares (Fig 1). She was ultimately managed with gabapentin 1200 mg 3 times daily, diphenhydramine 100 mg nightly, cetirizine 20 mg twice daily, and topical oxymetazoline cream, but still experienced weekly flares. Her quality of life was affected due to the persistent, unpredictable nature of flares, and she expressed a desire for better symptom control.

**Fig 2:**
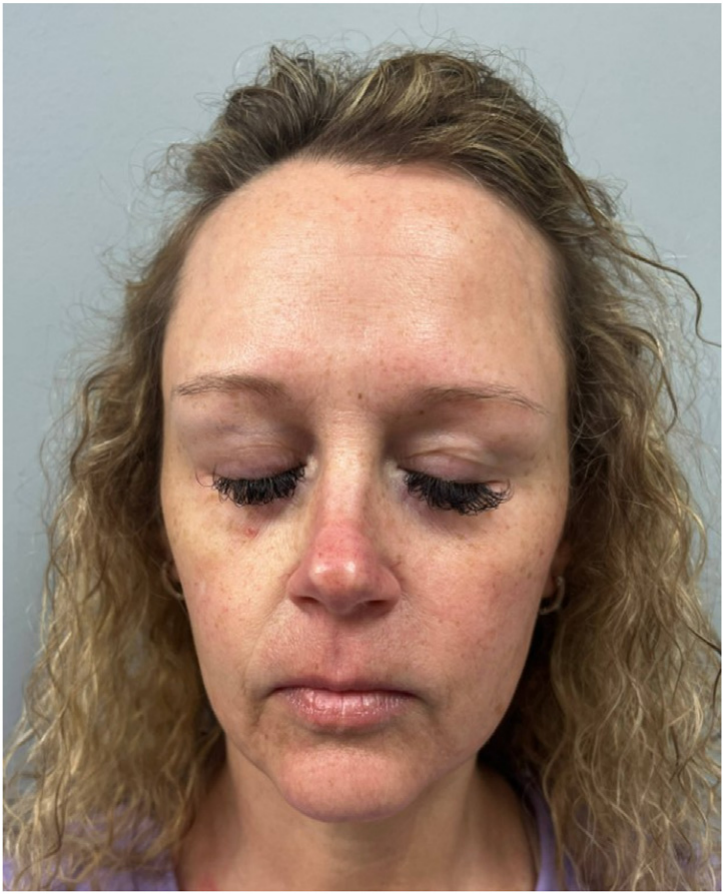
Patient at follow-up visit 4 weeks after receiving hyper-dilute BoTNA to the full face. *BoNTA*, Onabotulinum toxin A.

At a follow-up visit, the patient reported focal relief of erythromelalgia symptoms after receiving onabotulinum toxin A (BoNTA) injections to her glabella and frontalis for headaches. Thus, we decided to trial hyper-dilute BoNTA to the full face. Informed consent was obtained prior to treatment. We applied topical compounded ointment containing 15% lidocaine and 5% prilocaine to the treatment area for 30 minutes. Prior to injections, we removed the topical anesthetic and cleaned the skin with chlorhexidine gluconate. A 50-unit (U) vial of BoNTA was diluted with 5 mL saline for an aliquot of 1 U per 0.1 mL of solution. We injected the superficial dermis in a 1 cm grid pattern with 0.5 U per injection (0.05 mL/cm^2^) along the forehead, cheeks, upper lip, chin, and neck for a total of 50 U and 100 injected sites. The patient tolerated the procedure well with mild pinpoint bleeding. At her 1-month follow-up, she reported no adverse side effects post-treatment and, most remarkably, complete resolution of facial flares at 2 weeks post-treatment (Fig 2). At her 14-week follow-up, she reported continued resolution of facial flares.

## Discussion

Erythromelalgia has a complex pathophysiology with several neuromodulating substances involved in its onset of symptoms. Patients often require multiple concomitant therapies to achieve symptom control. Our patient received a multimodal treatment approach and had a reduction in flare frequency with drugs targeting serotonergic and histamine pathways (gabapentin, diphenhydramine, and cetirizine). The quickest and most effective response, however, was the use of hyper-dilute BoNTA to the full face.

We hypothesized that our patient’s symptom severity and frequency of painful flares would decrease with BoNTA injections due to her symptom relief on the forehead post-treatment for headaches. BoNTA provides symptom relief for other neuropathic pain conditions, although BoNTA has received U.S. Food and Drug Administration approval only for chronic migraine prophylaxis to date.^4^ BoNTA modulates pain via cleavage of SNAP-25, which blocks SNARE-mediated vesicle release of acetylcholine as well as of neurotransmitters associated with pain signaling, including CGRP and substance P.^5^ BoNTA-mediated SNAP-25 cleavage also interferes with TRPV1 capsaicin receptor, a cation channel associated with nociception. Research also suggests that the pathophysiology of erythromelalgia involves microvascular dysfunction in which vasodilation and increased permeability paradoxically cause tissue hypoxia and, consequentially, pain, erythema, and flushing.^1^ There is increasing evidence that rosacea shares similar neurovascular phenomena to erythromelalgia, in which the neurotransmitters CGRP and substance P are key players in its pathogenesis.^6^ In one study, dilute BoNTA was an effective treatment for erythematotelangiectatic rosacea measured by an increase in patient satisfaction after only 1 week of treatment.^6^

In our case, we selected hyper-dilute BoNTA to administer superficially near the dermal vascular plexus and to decrease the likelihood of unwanted facial muscle weakness or paralysis. The use of hyper-dilute BoNTA is a novel treatment for refractory erythromelalgia that effectively decreased symptom severity and frequency in our patient. While there are reported cases of BoNTA treatment of refractory erythromelalgia on the face and extremities,^7^, ^8^, ^9^ little is reported on its utility in a hyper-dilute form, which allows for a greater surface area of administration.

Hyper-dilute BoNTA is a novel and safe treatment for neuropathic pain and microvascular conditions such as erythromelalgia. This patient had rapid improvement in symptom severity and frequency in an otherwise refractory case of erythromelalgia; thus, we propose hyper-dilute BoNTA as another potential option for treatment.

## Conflicts of interest

None disclosed.
